# Social anxiety disorder and childhood trauma in the context of anxiety (behavioural inhibition), impulsivity (behavioural activation) and quality of life

**DOI:** 10.4102/sajpsychiatry.v25i0.1189

**Published:** 2019-01-28

**Authors:** Carolien J.W.H. Bruijnen, Susanne Y. Young, Melanie Marx, Soraya Seedat

**Affiliations:** 1 Centre of Excellence for Korsakoff and Alcohol-Related Cognitive Disorders, Vincent van Gogh Institute for Psychiatry, the Netherlands; 2 Donders Institute for Brain, Cognition and Behaviour, Radboud University Nijmegen, the Netherlands; 3 Nijmegen Institute for Scientist-Practitioners in Addiction, Radboud University Nijmegen, the Netherlands; 4 Department of Psychiatry, Faculty of Medicine and Health Sciences, Tygerberg Campus, Stellenbosch University, South Africa

## Abstract

**Background:**

Social anxiety disorder (SAD) is one of the most prevalent psychiatric disorders in South Africa. Previous studies have linked childhood trauma with the development of SAD. The behavioural inhibition system (BIS) and the behavioural activation system (BAS), two dimensions of personality related to anxiety and impulsivity, respectively, are said to influence the development of psychopathology, including SAD. Both SAD and childhood trauma have an impact on quality of life. This study investigated the relationship between BIS, BAS and quality of life in patients with SAD with and without exposure to childhood trauma, compared to healthy controls.

**Method:**

Data were collected for 102 adults. A total of 76 participants met SAD criteria, of which 51 were exposed to childhood trauma and 25 were not. The remaining 26 participants were demographically matched healthy controls. Measures of anxiety, impulsivity and quality of life were obtained by administering Carver and White’s BIS/BAS scales and the Quality of Life Enjoyment and Satisfaction Questionnaire – Self Report.

**Results:**

A positive correlation was found between the severity of SAD symptoms and the amount of childhood trauma exposure. No significant differences in impulsivity were found across the three groups. Healthy controls reported significantly lower anxiety and a better quality of life than both groups with SAD, while no differences were found between patients with SAD and childhood trauma and those without childhood trauma.

**Conclusion:**

More childhood trauma exposure appears to be associated with greater SAD severity. The lack of differences in BIS, BAS and quality of life in patients with SAD with or without childhood trauma requires further investigation.

## Introduction

With a lifetime prevalence of 15.8%, anxiety disorders are the most prevalent class of psychiatric disorders among South Africans, closely followed by substance use disorders (13.3%) and mood disorders (9.9%). The lifetime prevalence of social anxiety disorder (SAD) in adults is 2.8%.^1^ According to the Diagnostic and Statistical Manual of Mental Disorders, 5th Edition (DSM-5), characteristic symptoms of SAD include intense fear of embarrassment, humiliation and negative evaluation when in the company of unfamiliar people or when exposed to possible scrutiny by others in social situations. This usually leads to avoidance of these feared situations, which can, in turn, interfere with social and career functioning.^2^

Childhood trauma is associated with increased risk of developing psychopathology later in life. Swain et al.^3^ recently found a positive correlation between post-traumatic stress symptoms (related to childhood trauma) and anxiety in young adolescents. They concluded that childhood trauma may lead to social and emotional consequences later in life. Simon et al.^4^ found that childhood trauma, in particular emotional neglect, was associated with greater symptom severity in a population with SAD. In addition, exposure to childhood trauma had a significant negative effect on functioning, resilience and quality of life.

Research suggests that childhood trauma, in the context of SAD specifically, has desensitising effects on cortisol reactivity.^5,6,7,8^ Maeda et al.^6^ suggest that deficits in cortisol reactivity result in avoidance behaviours, leading to persistent fear responses, which, in turn, may play a crucial role in the psychopathology of social anxiety. Furthermore, Keyes et al.^9^ found an association between childhood trauma and an underlying liability to experience internalising (e.g. major depression and social phobia) or externalising (e.g. antisocial personality disorder and substance use) disorders. When this association was accounted for, the statistical significance of the association between specific traumatic events and any psychopathology was lost. Prevention of childhood trauma may, therefore, be critical in reducing the incidence of many common psychiatric disorders.^9^

Gray^10^ proposed two basic dimensions of personality: anxiety and impulsivity. Firstly, he described a behavioural inhibition system (BIS) which is sensitive to punishment, non-reward and novel stimuli. When the BIS is activated, it causes inhibition of goal-directed behaviour that is thought to be linked to higher anxiety. Secondly, he described a behavioural activation system (BAS) which is sensitive to non-punishment, reward and escape from punishment. When the BAS is activated, it causes goal-directed behaviour that is thought to be linked to higher impulsivity. There has been much investigation on both systems in relation to different forms of psychopathology. For example, Kasch et al.^11^ examined the relationship between major depressive disorder and the BIS and BAS, and found that patients who were depressed had lower BAS and higher BIS scores compared to healthy controls (HCs). Also, across the depressed group, lower BAS scores were associated with greater symptom severity and worse outcomes. Meyer et al.^12^ found a link between the BIS and depressive symptoms, but not between the BAS and manic symptoms, in patients with bipolar I disorder. Furthermore, Gomez et al.^13^ found that patients with generalised anxiety disorder had significantly higher anxiety scores (BIS), and significantly lower extraversion scores (BAS), compared to HCs.

Behavioural inhibition appears to constitute a specific marker of risk for social phobia in middle childhood or adolescence; however, longer term follow-up is needed to elucidate the full spectrum of risk conferred by behavioural inhibition in adulthood.^14^ A meta-analysis of longitudinal studies of children with behavioural inhibition found a more than sevenfold increased risk of developing SAD in later childhood and during adolescence.^15^ However, only a few studies to date have investigated the BIS and BAS in relation to SAD in adults. One of these studies, by Morgan et al.,^16^ investigated the role of approach and withdrawal in psychopathology. The authors presented 23 patients with SAD and 48 HCs with the Carver and White’s BIS/BAS scales^17^ and found that patients with SAD had higher BIS scores than controls. However, they found no significant group difference in total BAS scores. They concluded that these findings corresponded with Gray’s model of high BIS activity underlying anxiety and avoidant behaviour.^16^ A study by Lochner et al.^18^ investigated the influence of personality traits, as measured by the Temperament and Character Inventory,^19^ in patients with SAD. They compared 33 patients with SAD with 76 HCs and found that patients with SAD had significantly higher scores on harm avoidance, but significantly lower scores on novelty seeking and self-directedness, compared to HCs. To date, no investigation has been conducted on anxiety and impulsivity in patients with SAD in the context of childhood trauma.

Social anxiety disorder also impacts quality of life. Safren et al.^20^ found a significantly lower mean quality of life score in 44 patients with SAD, compared to the normative sample of Frisch et al.,^21^ as measured by the Quality of Life Inventory.^22^ A study conducted by Wittchen et al.^23^ found that 23.1% of 65 patients with SAD had their quality of life severely impaired and 24.6% were categorised as being markedly impaired in comparison to only 4.6% of either severely or markedly impairment in the HC group of the same number of subjects. Rapaport et al.^24^ found a similar rate of about 21.0% of 358 patients with SAD who had severe impairment in their quality of life. This finding was not as striking as their finding that 63.0% of 366 patients with major depressive disorder had quality of life impairment.

This study investigated the relationship between SAD and childhood trauma with regard to the BIS and BAS and quality of life. Firstly, we hypothesised that there would be a positive correlation between the level of exposure to childhood trauma and the severity of SAD symptoms. Secondly, we hypothesised that participants with SAD and childhood trauma would have higher BIS activity and lower BAS activity compared to individuals with SAD without childhood trauma, who we expected to have higher BIS activity compared to HCs. Thirdly, we hypothesised that participants with SAD and childhood trauma would report the lowest quality of life, followed by participants with SAD without childhood trauma and HCs, respectively.

## Methods

### Participants

A total of 102 participants were included in the study, of which 51 met criteria for SAD and childhood trauma (SAD+), 25 met criteria for SAD but not for childhood trauma (SAD−) and 26 participants were age- and gender-matched HCs.

### Procedure

After clearance was obtained, participants were recruited through referrals from psychiatrists and psychologists in the Western Cape (contact details of clinicians were provided by the Mental Health Group database at Tygerberg Campus, Stellenbosch University). Furthermore, the Cape Town Trauma Centre and the South African Depression and Anxiety Group (SADAG) assisted in recruitment through placement of advertisements in their monthly newsletters and contact with potential participants with SAD through their databases and support groups. Further recruitment methods consisted of convenience sampling through plasma screen advertisements and local anxiety disorder support groups in the Cape Town area. Contact information of individuals who showed an interest in participating was forwarded to a research assistant telephonically or through an email. All interested individuals were contacted by a research assistant via telephone or email and were screened with a standardised screening questionnaire. A consultation was scheduled for all eligible participants during which informed consent was obtained and a full diagnostic assessment using the Mini International Neuropsychiatric Interview^25^ was undertaken. Exclusion criteria were as follows: any personality disorder or intellectual disability, a previous diagnosis of any neurological disorder or any psychotic disorder according to DSM-5 criteria,^2^ and reported substance abuse or dependence (drug, alcohol, prescription and/or other). Cannabis users were included if they were completely abstinent for at least 2 weeks prior to study entry.

### Materials

Once the diagnostic assessment was completed, the participants were assessed for SAD, childhood trauma, impulsivity and anxiety (BIS and BAS) and quality of life with the following instruments:

1. **Liebowitz Social Anxiety Scale:**^26^ The Liebowitz Social Anxiety Scale (LSAS) is designed to assess the presence of SAD through 24 items that describe social situations that could cause anxiety. All items are assessed on two components, both rated on a scale of 0–3: fear or anxiety (‘none’ to ‘severe’) and avoidance (‘never, 0%’ to ‘usually, 67.0% – 100.0%’). To distinguish between SAD and HCs, a cut-off score of 60 was used. The LSAS has been found to be a reliable, valid and treatment-sensitive tool.^27^ The Cronbach’s alphas in the current sample were α = 0.94 for fear or anxiety, α = 0.92 for avoidance and α = 0.96 for the total scale.
2. **Childhood Trauma Questionnaire – Short Form:**^28^ The Childhood Trauma Questionnaire – Short Form (CTQ-SF) consists of 28 questions assessing five dimensions of childhood maltreatment: physical abuse, emotional abuse, sexual abuse, physical neglect and emotional neglect. Each question is answered on a Likert scale of 1 (‘never true’) to 5 (‘very often true’). Of the 28 items, three make up a minimisation or denial scale that is designed to detect false-negative trauma reports. A score of 0–1 on this scale is acceptable, whereas a score of 2–3 is indicative of response bias and results in exclusion.^29^ For categorisation according to severity, Bernstein et al.^30^ suggested the following cut-offs based on the total score: none to minimal trauma (25–36), low to moderate trauma (41–51), moderate to severe trauma (56–68) and severe to extreme trauma (73–125).^29^ For the purpose of this study, participants with scores between 25 and 40 were categorised as non-traumatised and participants with scores between 46 and 125 were categorised as traumatised. To be able to make a clear distinction between the two groups, participants scoring in the intermediate range (41–45) were also excluded. The Cronbach’s alphas in the current sample were α = 0.83 for physical abuse, α = 0.88 for emotional abuse, α = 0.92 for sexual abuse, α = 0.67 for physical neglect, α = 0.88 for emotional neglect and α = 0.92 for the total score, excluding the minimisation or denial scale.
3. **Carver and White's BIS/BAS Scales:**^17^ This scale consists of 24 items divided into the BIS and BAS scales. The BAS scale is subdivided into three factors: drive, fun seeking and reward responsiveness.^10^ Each question is answered on a Likert scale of 0 (‘very true’) to 4 (‘very false’). The Cronbach’s alphas in the current sample were α = 0.77 for BIS, α = 0.86 for BAS, α = 0.79 for BAS drive, α = 0.74 for BAS fun seeking, α = 0.70 for BAS reward responsiveness and α = 0.75 for the total scale, excluding the filler items. Poythress et al.^31^ and Vandeweghe et al.^32^ found similar Cronbach’s alphas in a sample of 1515 offenders.
4. **Quality of Life Enjoyment and Satisfaction Questionnaire – Self Report:**^33^ The Quality of Life Enjoyment and Satisfaction Questionnaire – Self Report (QLESQ-SR) consists of eight categories: physical health or activities, feelings, work, household duties, school or course work, leisure time activities, social relations and general activities. Each category has several statements that are assessed on a Likert scale of 1 (‘not at all’) to 5 (‘frequently or all of the time’), except for the last category that is answered in terms of satisfaction (1 = ‘very poor’ to 5 = ‘very good’). For the categories work, household duties and school or course work, a score of 0 indicates ‘does not apply’. The Cronbach’s alphas in the current sample were α = 0.93 for physical health or activities, α = 0.94 for feelings, α = 0.99 for work, α = 0.96 for household duties, α = 0.99 for school or course work, α = 0.88 for leisure time activities, α = 0.90 for social relations, α = 0.89 for general activities and α = 0.97 for the total score. Ritsner et al.^34^ found Cronbach’s alphas in roughly the same range.

### Statistical analysis

Firstly, missing values were imputed by calculating the participants’ mean score on the corresponding (subscales of the) questionnaire. Imputing missing values made all data complete, except for one scale of the QLESQ-SR, which was not answered by one participant. This participant was excluded from analyses involving the QLESQ-SR.

Patient characteristics are reported as appropriate. To examine any differences between groups (HC, SAD- and SAD+), chi-square tests (Monte Carlo adjusted Fisher’s exact tests, because of unequal groups, large contingency tables and sparse distribution of frequencies) with a two-tailed confidence interval of the *p*-value of 99.0% were used. The significance level was set at *p* (two-tailed) = 0.05. Correlations were calculated using Pearson’s correlations.

Multivariate analyses of variance (MANOVAs) were used to compare the BIS/BAS scale and BAS factors (drive, fun seeking and reward responsiveness), respectively, between groups (HC, SAD- and SAD+), with Bonferroni adjusted post-hoc test. Univariate analyses of variance (ANOVAs) were used to compare quality of life between groups (HC, SAD- and SAD+), for the eight-scale total of the QLESQ-SR and a five-scale total (excluding participants for whom work, household duties and/or school or course work did not apply). All data were analysed using SPSS version 24.0 for Windows.

## Ethical consideration

Permission to conduct the study and ethical clearance were obtained from the University of Stellenbosch Health Research Ethics Committee at Tygerberg Campus.

## Results

### Patient characteristics

All participants were between 20 and 72 years of age, with an average age of 34.25 years (standard deviation [s.d.] = 11.17), and an average of 14.74 years of education (s.d. = 3.08). There were 46 men (45.1%).

There were no significant group differences for patients with SAD and childhood trauma, patients with SAD without childhood trauma or HCs on the following variables: age (*F*_2,99_ = 2.49, *p* = 0.089), gender (χ^2^ = 0.21, *p* = 0.965), ethnicity (χ^2^ = 12.57, *p* = 0.061), marital status (χ^2^ = 5.54, *p* = 0.709), living arrangements (χ^2^ = 3.85, *p* = 0.714), employment (χ^2^ = 1.19, *p* = 0.562), being the breadwinner (χ^2^ = 0.11, *p* > 0.999) or yearly household income (χ^2^ = 10.07, *p* = 0.595). Next, there was no significant difference in comorbid anxiety disorders (χ^2^ = 0.75, *p* = 0.449) or mood disorders (χ^2^ = 0.67, *p* = 0.446) in patients with SAD with childhood trauma and those without childhood trauma. There was, however, a significant group difference for education (*F*_2,99_ = 3.96, *p* = 0.022) and post-hoc tests showed that HCs had significantly more years of education than patients with SAD and childhood trauma (*M*_diff_ = 1.89, *p* = 0.031; Table 1). All of the analyses that follow were therefore controlled for education.

**TABLE 1:** Demographic data of the total sample and per patient group.

Variables	Total (*N* = 102)		SAD- (*n* = 25)		SAD + (*n* = 51)		HC (*n* = 26)		*p*
	*n*	%	*n*	%	*n*	%	*n*	%	
Mean age in years (s.d.)	34.25	11.17	34.64	10.67	36.12	12.39	30.23	7.96	0.089
Mean education (s.d.)	14.74	3.08	15.28	3.54	13.92	2.51	15.81	3.31	0.022*
**Gender**									0.965
Female	56	54.9	13	52.0	28	54.9	15	57.7	
Male	46	45.1	12	48.0	23	45.1	11	42.3
**Ethnicity**									0.061
Black	10	9.8	-	-	7	13.7	3	11.5	
Mixed race	21	20.6	3	12.0	15	29.4	3	11.5
White	68	66.7	22	88.0	27	52.9	19	73.1
Asian	1	1.0	-	-	1	2.0	-	-
Other	2	2.0	-	-	1	2.0	1	3.8
**Marital status**									0.709
Single	57	55.9	13	52.0	28	54.9	16	61.5	
Married	26	25.5	7	28.0	13	25.5	6	23.1
With a partner	10	9.8	2	8.0	4	7.8	4	15.4
Divorced	7	6.9	3	12.0	4	7.8	-	-
Widowed	2	2.0	-	-	2	3.9	-	-
**Living arrangements**									0.714
Alone	25	24.5	6	24.0	14	27.5	5	19.2	
With family	43	42.2	8	32.0	24	47.1	11	42.3
With friends	12	11.8	4	16.0	5	9.8	3	11.5
With spouse or partner	22	21.6	7	28.0	8	15.7	7	26.9
Employed (yes, %)	67	65.7	17	68.0	31	60.8	19	73.1	0.562
Breadwinner (yes, %)	43	42.2	11	44.0	21	41.2	11	42.3	0.999
**Yearly household income**									0.595
Less than R10 000	8	7.8	3	12.0	3	5.9	2	7.7	
R10 000 – R20 000	3	2.9	-	-	2	3.9	1	3.8
R20 000 – R40 000	4	3.9	1	4.0	2	3.9	1	3.8
R40 000 – R60 000	8	7.8	-	-	7	13.7	1	3.8
R60 000 – R100 000	12	11.8	2	8.0	8	15.7	2	7.7
More than R100 000	66	64.7	19	76.0	28	54.9	19	73.1

s.d., standard deviation; SAD-, patients with social anxiety disorder without childhood trauma exposure; SAD+, patients with social anxiety disorder and childhood trauma exposure; HC, age- and gender-matched healthy controls; *, HC > SAD+.

### Social anxiety disorder and childhood trauma

The severity of SAD symptoms was significantly correlated with the level of reported childhood trauma (*r* = 0.42, *p* < 0.001).

### Social anxiety disorder and behavioural inhibition or behavioural activation

A significant main effect of group on total BIS and total BAS scores was revealed (*Λ* = 0.64, *F*_4,19_ = 12.36, *p* < 0.001). Tests of between-subject effects revealed a significant effect for BIS (*F*_3,98_ = 19.20, *p* < 0.001), but not for BAS (*F*_3,98_ = 2.56, *p* =0.059). Post-hoc tests further showed that HCs scored lower on the BIS than both patients with SAD without childhood trauma (*M*_diff_ = 5.16, *p* < 0.001), and patients with SAD and childhood trauma (*M*_diff_ = 4.80, *p* < 0.001). The difference between patients with SAD and childhood trauma and those without childhood trauma were not significant (*M*_diff_ = 0.37, *p* > 0.999). For the BAS, none of the differences were significant (HC vs. SAD−: *M*_diff_ = 4.41, *p* = 0.065; HC vs. SAD+: *M*_diff_ = 2.41, *p* = 0.462; SAD− vs. SAD+: *M*_diff_ = 2.00, *p* = 0.703). No significant main effect of group on the BAS factors was revealed (*Λ* = 0.94, *F*_6,19_ = 0.98, *p* = 0.438).

### Social anxiety disorder and quality of life

A significant effect of group on quality of life was found (*F*_3,97_ = 30.85, *p* < 0.001), and post-hoc tests further showed that HCs reported a significantly better quality of life than both patient groups (HC vs. SAD+: *M*_diff_ = 107.08, *p* < 0.001; HC vs. SAD−: *M*_diff_ = 98.04, *p* < 0.001). The difference between patients with SAD and childhood trauma and those without childhood trauma was not significant (*M*_diff_ = 9.04, *p* > 0.999).

To control for participants for whom work, household duties and/or school or course work did not apply, the same analysis was repeated without these scales. The main effect of group remained significant (*F*_3,97_ = 37.34, *p* < 0.001), and post-hoc tests did not differ much from the previous analysis. HCs still reported a significantly better quality of life than both patients with SAD without childhood trauma (*M*_diff_ = 61.71, *p* < 0.001) and those with childhood trauma (*M*_diff_ = 70.68, *p* < 0.001), and the difference between both patient groups was still not significant (*M*_diff_ = 8.97, *p* = 0.639).

## Conclusion and discussion

Little is known about SAD in the context of early childhood trauma. The literature shows that childhood trauma can have a great impact on the future of a child’s life.^4,5,9^ This study investigated the potential relationship between SAD and childhood trauma with regard to behavioural inhibition, behavioural activation and quality of life. In line with the literature, we found a strong positive relationship between SAD symptom severity and the extent of reported childhood trauma.^3,4,9,27,35^

With regard to behavioural inhibition, we hypothesised that patients with SAD and childhood trauma would score higher than patients with SAD without childhood trauma, and that HCs would report the lowest scores. Our findings replicate the findings of Morgan et al.^16^ in that our HCs had significantly lower BIS scores than patients with SAD with and without childhood trauma. Unexpectedly, we found no significant differences in BIS scores between patients with SAD and childhood trauma and those without childhood trauma. The lack of a statistically significant difference in BIS between the SAD groups suggests that early risk factors other than childhood abuse and neglect per se may be at play. One study found that exposure to early maternal stress, which was also partially mediated by childhood cortisol levels, predicted higher and more chronic inhibition and was associated with the development of SAD in adolescence.^36^ While the CTQ-SF measures a broad range of abuse and neglect experiences, it does not measure maternal stress. As such, early maternal stress, which may set children on a trajectory of high inhibition and evolution to SAD, was not evaluated here. Further research incorporating more broad-based measures of early adversities and stressors is needed to better understand the link between trauma, SAD and behavioural inhibition. As for behavioural activation, we hypothesised that patients with SAD and childhood trauma would score lower than patients with SAD without childhood trauma. In our data, we found no differences among these groups, suggesting that levels of impulsivity are not affected by SAD or childhood trauma.

In terms of perceived quality of life, both patients with SAD and childhood trauma and those without childhood trauma rated their quality of life significantly lower than HCs. These findings are in line with research by Safren et al.^20^ However, the lack of difference in quality of life scores between patients with SAD and childhood trauma and those without childhood trauma contradicts the findings of Simon et al.^4^ who found that childhood trauma had a negative effect on quality of life. Rapaport et al.^24^ mentioned a possible confounding factor in their study that may also be at play in the current study, namely that the early onset of SAD may have altered a patient’s view or belief about what is a normal quality of life. As such, patients with SAD may not have perceived their quality of life as compromised.

Limitations of the study include the presence of comorbidity, which may have confounded the findings. Participants with other psychiatric disorders (e.g. post-traumatic stress disorder, major depressive disorder or lifetime substance use disorder) were included, provided that SAD was the primary diagnosis. Yoon et al.^37^ examined the effects of comorbidity on cortisol stress reactivity in SAD and indicated that SAD is associated with increased cortisol stress reactivity, which is dampened by comorbid depression. This finding emphasises the need to consider participants’ comorbidity statuses and highlights the importance of clearly describing the characteristics of the samples used in research while reporting results. On the other hand, the exclusion of secondary comorbid disorders could also result in the exclusion of a significant number of psychiatric patients who have experienced childhood trauma. The literature shows that significant physical, emotional and sexual abuse and neglect in childhood can lead to the development of psychopathology (including anxiety disorders) later in life.^38,39^ Secondly, there is reason to believe that non-significant group findings may be a function of the small group sizes and other confounding factors in non-treatment-seeking individuals. It is likely that non-treatment-seeking individuals are better functioning than treatment-seeking individuals, accounting for fewer and less pronounced deficits.^40^

This was the first study to examine the contribution of childhood trauma to behavioural inhibition, behavioural activation and quality of life in patients with SAD. There is a clear and profound relationship between SAD symptom severity and childhood trauma exposure. Furthermore, anxiety, or behavioural inhibition, is more pronounced in patients with SAD compared to HCs; however, impulsivity, or behavioural activation, is not. Not surprisingly, HCs report a significantly better quality of life than individuals suffering from SAD, although patients with SAD who endured childhood trauma reported a slightly better quality of life than patients with SAD who were not exposed to childhood trauma, albeit non-significantly. It is possible that childhood trauma desensitises a child to what is ‘normal’ in life. Another possibility is that these individuals may have perceived their lives as being of better quality now compared to when they were children exposed to trauma. In conclusion, our research suggests that there is a relationship between SAD, an individual’s perceived quality of life and the level of experienced anxiety. The occurrence of childhood trauma in SAD did not significantly differ in impact on these aspects. This is not to say that childhood trauma does not pervasively impact SAD and other psychopathology – its prevention may be critical in reducing the incidence of many common psychiatric disorders, including SAD.^9^
